# Paternal Depression and Anxiety During the COVID-19 Pandemic

**DOI:** 10.3390/ijerph22010124

**Published:** 2025-01-19

**Authors:** Emily E. Cameron, Kayla M. Joyce, Kathryn Hatherly, Leslie E. Roos

**Affiliations:** 1 Faculty of Education, Simon Fraser University, 8888 University Drive, Burnaby, BC V5A 1S6, Canada; 2 Department of Psychology, University of Manitoba, 190 Dysart Road, Winnipeg, MB R3T 2N2, Canada; joycek1@myumanitoba.ca (K.M.J.); krollin4@uwo.ca (K.H.); leslie.roos@umanitoba.ca (L.E.R.); 3 Department of Clinical Health Psychology, University of Manitoba, 771 Bannatyne Avenue, Winnipeg, MB R3E 3N4, Canada; 4 School of Communications Science and Disorders, Western University, 1151 Richmond Street, London, ON N6A 3K7, Canada; 5 Department of Pediatrics and Child Health, University of Manitoba, 840 Sherbrook Street, Winnipeg, MB R3E 0Z3, Canada; 6 Children’s Hospital Research Institute of Manitoba, 715 McDermont Avenue, Winnipeg, MB R3E 3P4, Canada

**Keywords:** anxiety, COVID-19, depression, fathers, mental health, paternal

## Abstract

The COVID-19 pandemic significantly impacted the lives of families worldwide. Findings suggest a substantial impact of the COVID-19 pandemic on maternal mental health. Yet, much less is known about the impact of COVID-19 on paternal mental health. This study describes depression and anxiety and risk and protective factors among fathers of young children largely residing in Canada during the COVID-19 pandemic. Fathers (*N* = 70) of children 0 to 8 years old self-reported depression (EPDS, CESD, CESD-R) and anxiety (PASS, GAD-7) symptoms, while mothers (*N* = 236) provided reports of paternal depressive symptoms using the EPDS-P. Fathers evidenced clinically significant depression (37.1%) and anxiety (22.9%). Linear regression models with significant bivariate correlates indicated that depressive symptoms were associated with a mental health history and experiencing recent stressful events in the past month, while anxiety symptoms were related to mental health history over and above other correlates. Mother-reported paternal depression was prevalent (61.9%) and associated with lower mother-reported marital quality and higher maternal depressive symptoms. Of the fathers reporting clinically significant mental health concerns, less than one-third reported accessing mental health services. Paternal depression and anxiety symptoms were elevated during the pandemic, when compared to pre-pandemic comparisons. The risk and protective factors for paternal depression and anxiety included mental health history, recent stressful events, maternal marital quality, and maternal depressive symptoms. Longitudinal studies evaluating the persistent impact of the COVID-19 pandemic on paternal mental health are needed to inform continued efforts to repair the pandemic’s impact on family wellbeing.

## 1. Introduction

The COVID-19 pandemic affected families worldwide, as evidenced by a call-to-action to evaluate and promote parental well-being during the pandemic [1]. Early research from the pandemic underscores the impact that COVID-19-related restrictions and stressors had on maternal mental health, with three- to five-fold increases in the prevalence of maternal depression and anxiety [2,3]. Meta-analytic, systematic, and scoping review findings on maternal well-being have similarly highlighted the immense impact of the COVID-19 pandemic on maternal mental health [4,5,6,7], an impact that has persisted post-pandemic [8]. Comparatively, there is a dearth of research assessing the influence COVID-19-related factors have had on paternal mental health, although qualitative studies have highlighted substantial changes to the lives of fathers’ due to the pandemic [9,10]. The understudied area of paternal mental health is particularly troublesome, as results from previous quarantines and the COVID-19 pandemic indicate an increased mental health risk amongst caregivers [11,12]. It is therefore crucial to identify the quantitative extent to which COVID-19-related factors influenced paternal mental health to promote the development and implementation of appropriate interventions for the entire family as well as to inform post-pandemic research and clinical practice.

Paternal mental health concerns affect child developmental and mental health outcomes, negatively impact partner relationships and mental well-being, and impair overall family functioning [13,14,15]. For instance, children of fathers who have mental health disorders are at a higher risk of developing internalizing and externalizing disorders, as well as impaired social, emotional, and behavioral functioning [16,17]. Paternal depressive symptoms also increase the risk of marital dissatisfaction and poor marital quality [13,18]. In turn, marital dissatisfaction affects dyadic parenting behaviors (e.g., discipline, warmth/acceptance), decreases paternal–child interactions, and increases risk of poor child adjustment and child psychopathology [19]. Therefore, the effects of paternal mental health on children and partners may act individually or in concert to drive changes in family well-being. As such, a better understanding of paternal mental health during the pandemic is critical to providing effective and comprehensive measures to remediate poor family and child outcomes post-pandemic.

Meta-analyses and systematic reviews have highlighted the negative effects of the COVID-19 pandemic on adult mental health, with prevalence rates ranging from 15 to 33% and 16 to 31% for anxiety and depression, respectively [20,21,22]. Research suggests that parents were disproportionately affected by the COVID-19 pandemic compared to the non-parent adult population, due to the additional demands on home life with school and childcare closures as well as changes to family routines and activities [23]. Meta-analytic rates in perinatal maternal populations suggest pooled prevalence rates of 40% for prenatal anxiety, 27% prenatal depression, and 17% postpartum depression, with rates being significantly impacted by study location [5]. Mental health in perinatal mothers and mothers of young children up to age 8 years living predominantly in Canada showed similarly high rates of mental health concerns [2]. The limited research on fathers has largely focused on the perinatal period, with estimates ranging from 24 to 41% and 14 to 72% in prenatal and postpartum depression across Japan, China, and Sweden [24,25,26,27,28]. Given the limited findings and known impact of the pandemic on adults more broadly, investigating the effect of the pandemic on paternal mental health during sensitive periods of child development (i.e., perinatal and early childhood) is critical to prevent lasting impacts on children. Similarly, there were noted sex and gender differences in how COVID-19-related factors influenced parents, suggesting that mothers and fathers faced similar and distinct challenges which may have distinctly impacted their mental health. For instance, compared to their female counterparts, men experienced greater employment change from the onset of the pandemic [29], and, in turn, women experienced a faster pace of employment growth relative to men [30]. Thus, investigating the acute impact of the pandemic on fathers is important and provides information on risk for persistent effects of the COVID-19 pandemic.

For fathers, theoretical frameworks have highlighted the specific impact of COVID-19-related factors (e.g., role shifts in fatherhood, employment changes, financial strain, and reduced social functioning) on paternal mental health [31,32]. For instance, the transaction theory of stress and coping [33] hypothesizes that there is an interaction between multiple systems (e.g., cognitive, physiological, psychological) and an individual’s complex environment, which decreases the availability of factors that protect against depression and anxiety. Given the environmental and system changes for many families during the pandemic, there are many factors that may have impacted paternal mental health. For instance, the pandemic introduced a clear reduction in social support for individuals and families alike, which is a well-established risk factor for mental health concerns in fathers [34]. Additional important factors to evaluate include marital quality, family stressors (e.g., financial stress, recent stressful events), sociodemographic factors (e.g., employment), and mental health history, as these factors have been associated with depression and/or anxiety in fathers during key periods of change (i.e., the transition to parenthood) [34,35]. By evaluating the specific factors that increased or decreased risk for mental health concerns during the pandemic, tailored interventions can be developed to mitigate long-lasting mental health impacts on fathers who continue to struggle.

The current study aimed to replicate a previous study conducted with mothers, through a secondary data analysis of Cameron et al. [2], while extending findings to fathers, an understudied population in family research. We used a cross-sectional design to evaluate the prevalence of paternal depression and anxiety in fathers of young children as well as associated risk and protective factors.

## 2. Materials and Methods

### 2.1. Participants

A convenience sample was recruited between 14 April 2020 and 26 August 2020 for the Parenting During the Pandemic study. Recruitment strategies included social media, partnerships with community organizations, and indirect recruitment through knowledge translation mediums [2]. This study included a sample of men (*N* = 106) who identified as a father (e.g., biological, stepparent, adoptive parent) of a child ≤ 8 years old or who was expecting a child. Of the fathers participating in the study (*N* = 106), 70 fathers completed the entire survey. The original sample size of 106 participants came from fathers who started the survey, completed at least some of the questionnaires, but did not complete the entire survey. Only 70 fathers completed the whole survey. Partner reports from self-identified perinatal mothers (*N* = 236) were also used for paternal depression. The two samples (i.e., father and mother reports on fathers) comprised different subsets of families. With respect to inclusion/exclusion criteria, fathers had to be ≥18 years old to legally provide consent to participate (age of majority in Canada).

### 2.2. Procedure

Recruitment advertisements included a link to a study description. Prior to beginning the online survey, informed consent was obtained via the electronic data capture program (REDCap)—a metadata-driven methodology and workflow process for providing translational research informatics support [36,37]. Participants received an electronic copy of the informed-consent form via email. Once informed consent was complete, participants were re-directed to the online survey (see below for measure descriptions). Participants who completed the online survey were entered to win one of five electronic gift cards valued at $100 (Canadian dollars). The study protocol was approved by the University Research Ethics Board and adhered to the Declaration of Helsinki.

### 2.3. Measures

#### 2.3.1. Sociodemographic Information

Fathers were asked to identify the following socioeconomic information on behalf of their family: paternal age, marital status, parental education, gross annual family income, current residence location, and age of all children in the household. Information regarding household employment and current finances were also collected, including the impact of the COVID-19 pandemic on employment status. The father’s likelihood of applying for federal benefits and financial strain (e.g., ability to cover unexpected expenses) due to the COVID-19 pandemic was also gathered, where higher scores indicated greater financial strain.

#### 2.3.2. COVID-19

Author-compiled questions regarding COVID-19 were asked, including known exposure or vulnerability to COVID-19, if all individuals in the household were following social distancing guidelines, and how often members of the household were leaving their home for essential and non-essential services.

#### 2.3.3. Measures of Depression

Clinical cut-off scores of >10 for the Edinburgh Postnatal Depression Scale [38], ≥16 for the Center for Epidemiologic Studies Depression (CESD) and CESD—Revised [39,40], and ≥5 for the Edinburgh Postnatal Depression Scale—Partner [41] were used. These clinical cut-offs indicate the optimal identification of a depressive case, although they do not equate to a clinical diagnosis.

Edinburgh Postnatal Depression Scale (EPDS): For fathers of children 0 to 18 months old, depressive symptoms were measured using the 10-item EPDS [42]. An example item is “In the past 7 days, I have been so unhappy I have had difficulty sleeping”. Items are scored on a 4-point scale ranging from 0 to 3; several items are reverse-scored. Higher cumulative scores suggest more depressive symptoms. The EPDS is a valid measure of mood symptoms in fathers [38,43,44] and had acceptable internal consistency in the current study (α = 0.77).

Center for Epidemiologic Studies Depression (CESD) and CESD—Revised (CESD-R): For fathers of children between >1.5 to 8 years old, depressive symptoms were assessed using the 20-item CESD [40] or CESD-R [39]. An example item includes “During the last week, I was bothered by things that usually don’t bother me”. Both measures were scored using the original CESD scale, which ranges from ‘rarely or none of the time (less than one day)’ to ‘most or all of the time (5–7 days)’. Once several items are reverse-coded, responses are summed with higher total scores suggestive of greater depressive symptoms. Within the larger study [2], the first 351 participants completed the CESD, while the remainder completed the CESD-R. Both scales will be referred to as CESD hereafter. The CESD is a valid measure of depressive symptoms [45,46]. Internal consistency was good-to-excellent (α = 0.90–0.95).

Edinburgh Postnatal Depression Scale—Partner (EPDS-P): Mother reports of paternal perinatal depression were also available on the 10-item EPDS-P [41]. An example item is “In the past week, my partner has mentioned looking forward to enjoyment in things”. Items are scored on a 4-point scale ranging from 0 to 3, and several items are reverse-scored. Higher summative scores suggest more depressive symptoms. The EPDS-P is a valid measure of partner depressive symptoms [47] and good internal consistency was found in the current study (α = 0.82).

#### 2.3.4. Measures of Anxiety

A clinical cut-off score of ≥26 for the Perinatal Anxiety Screening Scale (PASS) [48] and ≥10 for the Generalized Anxiety Disorder -7 Item (GAD-7) [49] was used to signify clinically significant anxiety. Data on mother-reported paternal anxiety were not administered here, as there are no partner-reported anxiety measures which originated from the PASS or GAD-7.

Perinatal Anxiety Screening Scale (PASS): For fathers of children 0 to 18 months old, anxiety was measured using the 31-item PASS [48]. An example item includes “How often have you experienced worry about the baby/pregnancy?” Items are scored on a 4-point scale ranging from ‘not at all’ (=0) to ’almost always’ (=3), with higher summative scores suggesting greater perinatal anxiety. The PASS is a valid measure of perinatal anxiety in mothers [48], but research has not examined the validity of the PASS for fathers. Internal consistency was excellent in the current study (α = 0.96).

Generalized Anxiety Disorder—7 Item (GAD-7): For fathers of children >1.5 to 8 years old, anxiety symptoms were assessed using the GAD-7 [49]. An example item includes “Over the last two weeks, how often have you be bothered by feeling nervous, anxious, or on edge?” Items are scored on a 4-point scale ranging from “not at all” (=0) to “nearly every day” (=3), with higher summative scores indicating elevated anxiety symptoms. The GAD-7 demonstrates good validity [50,51] and had excellent internal consistency in the current study (α = 0.94).

#### 2.3.5. Multidimensional Scale of Perceived Social Support (MSPSS)

The 12-item MSPSS [52] assessed paternal perceived social support. An example item includes “There is a special person who is around when I am in need”. Items were scored on a 7-point scale ranging from “very strongly disagree” (=1) to “very strongly agree” (=7). Higher summative scores are indicative of higher levels of perceived social support. The MSPSS is a valid measure of perceived social support [53] and had excellent internal consistency in the current study (α = 0.94).

#### 2.3.6. Revised Dyadic Adjustment Scale (RDAS)

The 14-item RDAS [54] examined relationship quality. Participants were asked to rate agreement/disagreement with their partner on several items, such as “religious matters” and “demonstrations of affection”. Items are scored on a 5-point scale ranging from ‘always disagree’ (= 0) to ‘always agree’ (=5) and are subdivided into three subscales, including consensus, satisfaction, and cohesion. Lower summative scores suggest greater relationship distress. The RDAS is a valid measure [54] and showed good internal consistency in the current study (α = 0.83).

#### 2.3.7. Recent Stressful Experiences (RSE)

Based on expert recommendations from the Center on the Developing Child at Harvard University, the author-compiled RSE was used to assess the presence of stressors within the past month (‘RES past month’) and the past 2–12 months (‘RES past year’). Examples of recent stressful experiences include a life-threatening illness/accidental injury to you or someone close to you, family violence/abuse to you or someone close to you, and death of someone close to you. Items were scored dichotomously as 0 = No and 1 = Yes. Scores were summed to calculate a cumulative number of recent stressful experiences.

### 2.4. Statistical Analysis

Statistical analyses were conducted using IBM SPSS Statistics Version 25. Little’s Missing Completely at Random (MCAR) [55] indicated that missing data on the EPDS, CESD, PASS, and GAD-7 were MCAR (*p* > 0.500), while the EPDS-P was not MCAR (χ^2^ = 66.62, *p* < 0.001). EPDS-P missing data were imputed (*n* = 10) using expectation maximization. The regression produced nearly identical predictors with imputed and raw data; original raw data are reported.

Descriptive analyses were conducted to assess sample characteristics and average scores on independent and dependent variables. Father-reported depressive and anxiety symptoms were dichotomized based on clinical cut-off scores (see measures above) and combined into a single measure for depression (using the EPDS and CESD) and anxiety (using the PASS and GAD-7). Continuous variables for depression (EPDS, CESD) and anxiety (PASS, GAD-7) were converted into z-scores, which are standardized variables with a mean of 0 and standard deviation of 1 [56], to allow for these variables to be combined into a single continuous measure for both depression and anxiety [57]. Bivariate correlations were conducted to identify significant relationships between relevant variables and continuous combined scores. Significant correlations with outcome variables then informed linear regression to examine risk and protective factors for paternal depression and anxiety. Sample sizes of 29 and 85 were required for 80% power for correlation analyses to detect large and medium effect sizes, respectively; sample sizes of about 25 and 40 were required to detect a large and medium effect size (respectively) for linear regression analyses [58,59].

## 3. Results

### 3.1. Participant Characteristics

Fathers (*N* = 106) were 37.25 years old (*SD* = 6.31) and parenting 1.62 (*SD* = 0.77) children on average (see Table 1). Participants were largely residing in Canada (75.5%) or the United States (19.8%) and married or common-law (93.4%). Most fathers had at least a bachelor’s degree (62.9%) and an annual income > $100,000 (57.0%). Nearly all fathers (98.1%) had not experienced a diagnosis of COVID-19 within their household, but 25.5% reported knowing someone personally who had been diagnosed with COVID-19. Financial strain due to the pandemic was prevalent (38.7%), with 32.1% reporting that someone in the household had been laid off or lost at least half of their regular work hours. The prevalence of self-reported paternal mental health history was 44.3%. Nearly half of the sample reported experiencing at least one stressful event in the past month (47.1%) or past year (52.8%).

### 3.2. Prevalence and Correlates of Depression

**Figure 1:**
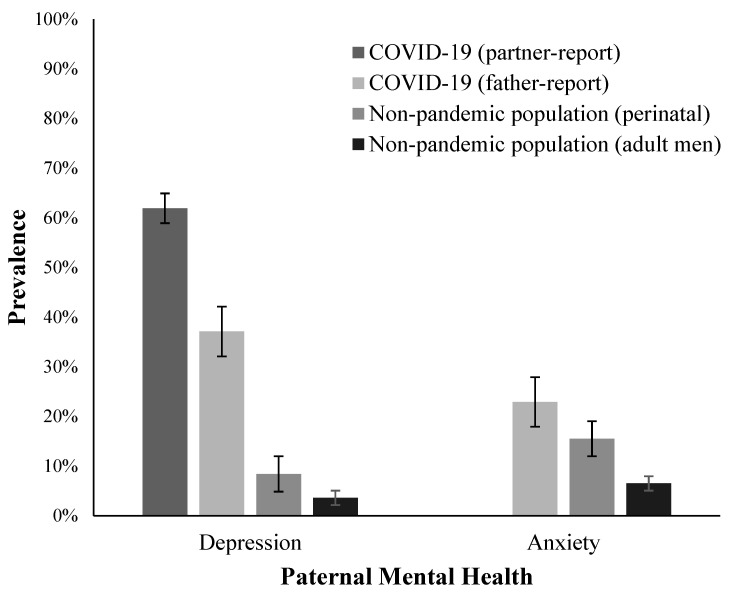
Prevalence rates of depression and anxiety during the COVID-19 pandemic with standard error bars and pre-pandemic comparisons. Note: Pre-pandemic estimates were drawn from the extant literature on paternal depression and anxiety. These samples represent different samples than the participants included in the current study [60,61,62,63].

Total depression prevalence above respective clinical cut-off scores was 37.1% (*n* = 26/70; Figure 1). Of fathers with a child 0 to 18 months old, 58.3% (*n* = 7/12) scored ≥ 10 on the EPDS (*M* = 10.50, *SD* = 4.08; Figure 1). Of fathers with a child > 1.5 to 8 years old, 32.8% (*n =* 19/58) scored ≥ 16 on the CESD (*M* = 13.50, *SD* = 13.93; Figure 1). Rates of elevated depressive symptoms did not differ across age groups d (χ^2^ = 2.79, *p* = 0.095).

Higher depressive symptoms were correlated with having a mental health history, lower total marital quality (and RDAS consensus and cohesion subscales), greater recent stressful events in the past month, greater financial strain, and lower household income during the COVID-19 pandemic (Table 2). In a linear regression model, household income (*t* = −0.60, *p* = 0.555), financial strain (*t* = −0.49, *p* = 0.628), and marital quality (*t* = −1.17, *p* = 0.247) were not significantly related to depressive symptoms over and above recent stressful events in the past month (*t* = 2.53, *p* = 0.015) and mental health history (*t* = 2.76, *p* = 0.009) (Table 3).

### 3.3. Corroboration from Maternal Reports of Paternal Depression

For fathers with a partner who was expecting or up to 18 months post-partum, the mothers completed the EPDS-P (*N* = 236), which assessed paternal depression. The prevalence of depressive symptoms above the clinical cut-off score of ≥5 was 61.9% (*n* = 146/236; *M* = 6.36, *SD* = 4.64), consistent with fathers’ self-reported perinatal depression prevalence of 58.3% (Figure 1).

Mother-rated paternal depression was significantly associated with higher maternal depression and anxiety, lower total maternal marital quality (and RDAS satisfaction and cohesion subscales), lower social support, recent stressful events in the past month and year, employment loss, financial strain, and household income (Table 2). In a linear regression model, household income (*t* = −1.18, *p* = 0.239), financial strain (*t* = 0.80, *p* = 0.428), employment loss (*t* = −0.10, *p* = 0.919), recent stressful events in the past month (*t* = 0.51, *p* = 0.613) or year (*t* = 0.24, *p* = 0.814), social support (*t* = 0.21, *p* = 0.837), and maternal anxiety (*t* = 0.91, *p* = 0.365) were not significantly related to depressive symptoms over and above maternal depressive symptoms (*t* = 2.68, *p* = 0.008) and maternal marital satisfaction (*t* = −2.25, *p* = 0.026) (Table 3).

### 3.4. Prevalence and Correlates of Anxiety

In the total sample, 22.9% (*n* = 16/70) of fathers met clinical cut-off scores for anxiety (Figure 1). Anxiety prevalence rates on the PASS (score ≥ 26) was 33.3% (*n* = 4/12) for fathers with a child between 0 and 18 months old (*M* = 19.86, *SD* = 17.50; Figure 1). For fathers with a child > 1.5 to 8 years old, anxiety prevalence on the GAD-7 (score ≥ 10) was 20.7% (*n* = 12/58; *M* = 5.97, *SD* = 6.26; Figure 1). Rates did not differ across age groups (χ^2^ = 0.90, *p* = 0.342).

Higher anxiety symptoms across age groups were associated with having a mental health history, lower total marital quality (and RDAS cohesion subscale), greater recent stressful events in the past month, and greater financial strain during the COVID-19 pandemic (Table 2). In a linear regression model, financial strain (*t* = −0.04, *p* = 0.969), recent stressful events in the past month (*t* = 1.86, *p* = 0.070), and marital quality (*t* = −0.85, *p* = 0.399) were not significantly related to anxiety symptoms over and above mental health history (*t* = 2.36, *p* = 0.023) (Table 3).

### 3.5. Mental Health Service Use

Of the fathers indicating unmet mental health needs, few fathers reported accessing individual (*n* = 3/16; 18.8%) or group counseling services (*n* = 1/14; 7.1%). A larger percentage of fathers sought mental health information online (*n* = 5/16; 31.3%) or used well-being Apps (*n* = 5/16; 31.3%). No fathers reported accessing instant messaging mental health services, a mental health crisis line, or faith-based counseling services.

## 4. Discussion

To our knowledge, this study was the first to assess the prevalence of depression and anxiety among primarily Canadian fathers with young children aged 0-8 years during the COVID-19 pandemic. Findings highlight that fathers faced significant mental health concerns during the first wave of the COVID-19 pandemic. Clinically significant depression (37.1%) and anxiety (22.9%) were prevalent among the entire sample. Perinatal fathers may have experienced even greater mental health need, with 58.3% meeting clinically significant cut-offs for depression and 33.3% for anxiety. Self-reported household income < $60,000, a history of mental health concerns, financial strain due to the pandemic, recent stressful events in the past month, and lower marital quality were identified as risk factors for paternal depression during the COVID-19 pandemic. Based on mother-reported paternal depression, higher maternal depression and lower maternal marital quality were associated with paternal depression. A history of mental health concerns, financial strain, recent stressful events in the past month, and lower paternal marital quality were independent risk factors for the development of paternal anxiety during the COVID-19 pandemic. Finally, few fathers reported using mental health services despite high rates of depression and anxiety.

The limited research on paternal mental health concerns during the pandemic evidences mixed findings on the impact of the COVID-19 pandemic on paternal well-being. Current findings are consistent with rates of prenatal and postpartum depression in some countries during the pandemic, such as 24–41% and 21–38% in prenatal and postpartum Japanese fathers [24,25]. Yet, these findings appear lower than prevalence rates documented in other countries, such as the United States (i.e., 72% of fathers met clinical cut-off for postpartum depression during the pandemic) [26]. Conversely, prevalence rates may be higher than expected compared to emerging data from other countries (e.g., 14% and 21% of fathers experienced postpartum depression in China and Sweden, respectively) [27,28]. Since rates of depression in fathers, especially perinatal fathers, has been shown to differ significantly across continents [60], we would expect discrepant findings. Perhaps these differences in paternal mental health concerns may be due to substantial differences in how countries responded to the COVID-19 pandemic and related access to perinatal care (e.g., [64]).

When comparing findings from the current study to Canadian pre-pandemic prevalence rates, results suggest the rates of depression and anxiety in Canadian fathers of young children increased during the first wave of the pandemic, based on observational comparisons of statistics of variance (e.g., confidence intervals, standard deviation) in the current study and related research. Rates of depression among perinatal fathers was 8.4% pre-pandemic [60], while the 12-month prevalence of depression in Canadian adult men was 3.6% [62]. Similarly, the prevalence rates of anxiety in the Canadian pre-pandemic population were estimated at 15.5% [61] and 6.5% [63] in perinatal fathers and adult men, respectively. These pre-pandemic comparisons suggest a 4- to 10-fold increase in depression and 1.5- to 3-fold increase in anxiety among fathers during the pandemic, consistent with the literature on COVID-19 [65]. Thus, Canadian fathers’ adjustment to a time of immense global uncertainty was reflected in unmet mental health needs, consistent with rates of mental health concerns in other countries during this time (e.g., [25,26]). This finding, in combination with similar support for a family-centered approach to post-pandemic mental healthcare [6,7,24,25], illustrates a critical need to promptly address mental health concerns through psychological interventions to avoid long-term implications of the COVID-19 pandemic on paternal mental health and family well-being. Findings have additional implications of informing future periods of global stressors and the impact on the family system.

Within the current sample of fathers, depression and anxiety were linked to a history of mental health concerns, lower household income, financial strain, recent stressful events in the past month, and lower marital quality. These findings are consistent with maternal correlates of depression and anxiety previously reported elsewhere [2] and the available data on fathers in other countries (e.g., [24]). The epigenetic hypothesis of mental illness [66] may help explain the mechanism by which pandemic-related stressors triggered a re-occurrence of mental health concerns. The epigenetic hypothesis of mental illness [66] posits that severe environmental stressors, such as the stressors associated with the COVID-19 pandemic, may trigger changes at the genetic level, which contributes to depression amongst at-risk individuals. Similarly, as previously described, the transaction theory of stress and coping [33] also explains the interaction between complex systems and one’s environment. This theory indicates that during the pandemic, COVID-19-related stressors may have increased one’s susceptibility toward developing mental health concerns, specifically among those who were already at risk (i.e., those who had a history of mental health concerns). Yet, in linear models for paternal depression, recent stressful events in the past month (in addition to a history of mental health concerns) were significantly associated variables over and above the other correlates. This finding suggests that fathers dealing with life stressors (e.g., death of a loved one, relationship break up, job loss, caring for ill family members, relationship abuse) in addition to the stress of the pandemic were at even greater risk for depression. Cumulatively, the epigenetic hypothesis of mental illness [66] and the transaction theory of stress and coping [33] provide strong theoretical evidence to highlight the increased vulnerability some fathers faced during the COVID-19 pandemic and the greatest risk for those navigating additional stressful events. Findings underscore the need to screen and monitor fathers with a history of mental health concerns, fathers with lower household income or at risk for financial strain, and couples to assess marital quality to provide early intervention and avoid the long-term detrimental impacts on fathers, their children, and the family unit. It is especially imperative to screen for additional life stressors that may provide a cumulative risk for poor mental health outcomes in the context of large-scale environmental stressors, such as the COVID-19 pandemic. These findings also suggest the need for policies to improve financial strain during large-scale crises, such as the related COVID-19 financial relief efforts (e.g., Canada Emergency Wage Subsidy, Canada Emergency Response Benefit), and increased access to financially prohibitive mental health services during and following these events to support the families.

In addition to self-reported depression, paternal depression was assessed via partner reports. Findings suggested that lower maternal marital quality and higher maternal depression are associated with higher paternal depressive symptoms. Results are consistent with previous reports of an inverse relationship between marital quality and maternal mental health [2] and provide clinical evidence that strengthening the marital relationship may be a key factor in alleviating parental depression, consistent with couple-focused treatment approaches [67]. The association between maternal and paternal depression is also consistent with the literature and the well-documented relationship between parental mental health in meta-analytic studies [60]. Timely and effective screening for mental health concerns in perinatal families is critical for identifying parents in need of support before mental health concerns become chronic. While screening guidelines for perinatal appointments specifically are key, additional screening measures may also be instrumental in identifying parental mental health concerns, such as integrating parental screening measures into child appointments and interventions [68]. It is also recommended that the EPDS-P be used as a screening tool to capture the depressive symptoms in both parents when only one is present at an appointment; the EPDS-P can be used for mothers or fathers as informants on the other parent [47]. Early intervention for depression in either parent is important to prevent the development of depression in the other parent. Early intervention for any parent with perinatal depression is crucial for preventing poor child developmental outcomes and subsequently ensuring children enter their school years ready to thrive [69]. Further, families with either parent experiencing depression should be considered at higher risk for worse outcomes and high priority for early intervention [69].

Our research also evidenced a lack of fathers (*N* = 70) completing research on parental mental health. For example, Cameron et al. [2] had 641 mothers complete their study on parenting during the pandemic within three weeks, relative to the 70 fathers who completed the same survey between 14 April 2020 and 26 August 2020. Findings suggest that fathers were less likely to complete studies on their mental health during the pandemic, consistent with the mental health literature suggesting that men are less likely to participate in research on mental health and to receive mental health services [70]. This finding may in part explain the limited research available on fathers since 2020; whereas, there is significantly more known about mothers and mother–child dyads. Efforts towards inclusive research and family-centered care continue to be critical for supporting the whole family system and promoting child development more comprehensively.

### 4.1. Strengths and Limitations

The findings of this study should be interpreted with several limitations in mind. First, results are based on cross-sectional data collected between 14 April 2020 and 26 August 2020, which provide a snapshot of paternal mental health during a period of acute stress and uncertainty. Future research should assess the influence of time since the onset of the COVID-19 pandemic on paternal depression and anxiety to evaluate differential emotional consequences. Results may also not generalize to fathers within lower socioeconomic classes, given the above-average reported household income for Canadian families (e.g., 53% reported a household income of >$100,000 versus the $61,400 Canadian average household income) [71]. Additionally, fathers were included in each appropriate child age range analysis, which allowed for classification of fathers with multiple children. This data analysis method increases the clinical utility of findings despite requiring some fathers to be included twice across analyses. Sample size was also disproportionate between mothers and fathers in the analyses, likely resulting in differences in statistical power and type I error rate across these analyses. Finally, it should be noted that psychometric properties of the PASS have not been described for fathers to date and self-reported levels of depression and anxiety are not equivalent to a clinical diagnosis.

Despite these limitations, this study focuses on an understudied population within the larger context of family well-being during a significant world-wide stressor. In addition to the novelty of this study, a strength of this research was the use of partner-rated measures for paternal depression, given the generally lower research participation of fathers. Further, while the majority of research on paternal mental health has primarily focused on the perinatal period [26,27,28], our sample included fathers of children up to age 8 years to provide a more comprehensive sample of fathers with children at sensitive periods of socioemotional development. While the focus of this work is on the first wave of the pandemic, compared to mothers, there is still limited research available on the impact of the pandemic for fathers specifically. The current study therefore provides important insights into the mental health of fathers to inform future research and clinical directions.

### 4.2. Clinical Implications

Prior to the pandemic, there was a clear need for interventions to support paternal mental health during key stages of changes [16,72]. With emerging research on the impact of the COVID-19 pandemic on fathers’ well-being, there is an immediate need for interventions that address paternal mental health post-COVID-19 pandemic to prevent children’s exposure to fathers with chronic and untreated mental health concerns. Cognitive behavioral therapy (CBT) is widely recognized as an efficacious intervention with a strong evidence-base for the treatment of a variety of disorders, including depression and anxiety [73,74]. Transdiagnostic psychological interventions, such as the CBT-based Unified Protocol [75] or Dialectical Behavior Therapy [76], that target underlying mechanisms of both depression and anxiety may be critical to mitigating overall mental health concerns in fathers. An additional consideration for therapeutic intervention is family and/or couple-based interventions that may increase maternal marital quality and subsequently improve both maternal [2] and paternal depression. Since the pandemic, there has been a substantial increase in digital mental health interventions (e.g., app-based, online, telehealth), which may be especially helpful to fathers, as most fathers seeking mental health support did so through self-directed online informational searches. Future research is needed on the specific supports that would benefit fathers with persistent mental health concerns.

## 5. Conclusions

In this cross-sectional study, fathers (like mothers) reported high prevalence rates of clinically significant depression and anxiety during the initial phase of the COVID-19 pandemic. Research with fathers during and post-pandemic continue to be mixed by study country. Our results provide evidence to encourage continued investigations in Canada, as well as internationally, to obtain a more comprehensive understanding of the experiences and continued impact of the pandemic on paternal mental health and subsequent father-child interactions.

## Figures and Tables

**Table 1:** Participant characteristics by respondent.

Characteristic		Father-Report (*N* = 106)		Mother-Report (*N* = 236)	
		%	Valid *N*	%	Valid *N*
Parent Education Level			105		236
	Some high school	1.0	1	1.3	3
	High school diploma or equivalent	14.3	15	8.1	19
	College/Technical School	21.9	23	17.8	42
	Bachelor’s degree	24.8	26	31.4	74
	Above bachelor’s degree	38.1	40	41.5	98
Employment Status during COVID-19			100		216
	Hours consistent	41.0	41	42.6	92
	More than half of regular hours	13.0	13	9.7	21
	Less than half of regular hours	10.0	10	10.2	22
	Laid off	11.0	11	18.1	39
	Salaried	25.0	25	19.4	42
Total Annual Household Income			100		220
	≤$20,000	2.0	2	2.3	5
	$20,001–$40,000	8.0	8	5.9	13
	$40,001–$60,000	4.0	4	7.3	16
	$60,001–$80,000	16.0	16	11.4	25
	$80,001–$100,000	13.0	13	13.2	29
	>$100,000	57.0	57	60.0	132
Marital Status			106		233
	Married/Common Law	93.4	99	96.1	224
	Divorced/Separated	1.9	2	0.0	0
	Single (never married)	4.7	5	3.9	9
Number of children			106		230
	0	1.9	2	0.4	1
	1	45.3	48	45.2	104
	2	45.3	48	40.4	93
	3+	7.5	8	13.9	32
Identifies in a Vulnerable Population			106		231
	Underlying medical condition	3.8	4	9.1	21
	Compromised immune system	5.7	6	6.1	14
Partner in a Vulnerable Population			105		234
	Underlying medical condition	5.7	6	8.1	19
	Compromised immune system	6.7	7	1.7	4
Child in a Vulnerable Population			106		235
	Underlying medical condition	4.7	5	4.7	11
	Compromised immune system	1.9	2	2.6	6
Medical Services Impacted by COVID-19			103		235
	Yes	34.0	35	57.9	136

**Table 2:** Bivariate correlations of sociodemographic and predictor variables for mothers and fathers of children age 0–8 years.

Measure	1	2	3	4	5	6	7	8	9	10	11	12	13	14	15	16	17	18
1. Depression	-	0.87 **	-	0.43 **	−0.11	−0.36 **	−0.35 *	−0.26	−0.29 *	0.33 **	0.19	0.24	0.28 *	−0.01	−0.01	−0.04	−0.24	−0.25 *
2. Anxiety	0.79 **	-	0.87 **	0.41 **	−0.10	−0.28 *	−0.24	−0.13	−0.28 *	0.26 *	0.09	0.16	0.31 *	0.08	0.01	0.09	−0.23	−0.14
3. EPDS-P *	0.45 **	0.42 **	-	-	-	-	-	-	-	-	-	-	-	-	-	-	-	-
4. MH history	0.29 **	0.31 **	0.08	-	−0.08	−0.15	−0.10	−0.07	−0.15	0.01	0.16	−0.14	0.12	0.07	0.02	0.30 *	−0.01	−0.09
5. MSPSS	−0.23 **	−0.15 *	−0.15 *	−0.08	-	0.30 *	0.32 *	0.39 **	0.08	−0.43 **	−0.17	0.15	−0.07	−0.37 **	0.26 *	0.09	0.10	−0.09
6. RDAS total	−0.34 **	−0.29 **	−0.23 **	−0.22 **	0.23 **	-	0.90 **	0.82 **	0.77 **	−0.40 **	−0.20	−0.18	−0.33 *	−0.36 **	−0.06	−0.18	−0.13	−0.04
7.RDAS consensus	−0.22 **	−0.22 **	−0.12	−0.11	0.15 *	0.80 **	-	0.77 **	0.46 **	−0.39 **	−0.32 *	−0.14	−0.22	−0.18	−0.04	−0.12	−0.02	−0.07
8. RDAS satisfaction	−0.42 **	−0.35 **	−0.30 **	−0.22 **	0.23 **	0.75 **	0.42 **	-	0.38 **	−0.43 **	−0.16	−0.27	−0.33 *	−0.37 **	−0.02	−0.12	−0.15	−0.13
9.RDAS cohesion	−0.21 **	−0.16 *	−0.15 *	−0.21 **	0.15 *	0.81 **	0.42 **	0.45 **	-	−0.20	−0.01	−0.00	−0.27	−0.34 *	−0.10	−0.21	−0.17	0.07
10. RSE past month	0.29 **	0.27 **	0.26 **	0.24 **	−0.10	−0.13	0.04	−0.19 **	−0.11	-	0.59 **	0.33 **	0.18	0.13	−0.12	−0.13	−0.13	0.02
11. RSE past year	0.17 **	0.17 **	0.16 *	0.16 *	−0.04	−0.05	0.08	−0.06	−0.13	0.43 **	-	0.09	0.23 *	0.02	−0.10	0.07	−0.01	−0.08
12. Employment Loss	0.17 **	0.16 *	0.17 *	0.08	−0.10	−0.06	−0.00	−0.04	−0.08	0.30 **	0.03	-	0.12	−0.06	−0.09	−0.33 **	0.03	−0.21 *
13. Financial Strain	0.24 **	0.23 **	0.23 **	0.01	−0.14 *	−0.08	−0.01	−0.10	−0.05	0.15 **	0.15 **	−0.21 **	-	−0.02	−0.02	0.11	−0.13	−0.16
14. Parent Age	−0.17 **	−0.15 *	−0.06	−0.08	0.10	−0.06	−0.04	0.01	−0.08	−0.16 **	−0.15 **	−0.09	−0.19 **	-	0.03	0.18	0.23 *	0.32 **
15. Marital Status	−0.02	0.02	−0.02	0.01	0.06	0.12	0.11	0.14 *	0.06	−0.12 *	−0.10	−0.06	−0.17 **	0.07	-	0.17	0.09	0.16
16. Parity	0.01	−0.05	−0.13	0.03	−0.13 *	−0.12	−0.13	−0.03	−0.04	0.06	−0.02	0.00	−0.02	0.12 *	0.01	-	0.05	0.17
17. Parent Education	−0.11	−0.11	−0.01	−0.14 *	0.16 **	0.11	0.06	0.10	0.06	−0.21 **	−0.14**	−0.16 **	−0.19 **	0.27 **	0.10	−0.19 **	-	0.12
18. Household Income	−0.14 *	−0.14 *	−0.14 *	−0.13 *	0.17 **	0.05	0.03	0.15 *	−0.05	−0.22 **	−0.11 *	−0.16 **	−0.25 **	0.26 **	0.21 **	−0.11 *	0.25 **	-

*Note.* Above the diagonal = father report; Below the diagonal = mother report. Abbreviations: EPDS-P = Edinburgh Postnatal Depression Scale—Partner (mother-reported paternal depression); MH = mental health; MSPSS = multidemensional scale of perceived social support; RDAS = revised dyadic adjustment scale; RSE = recent stressful event. * *p* <0.05, ** *p* < 0.01.

**Table 3:** Linear regression for paternal depression and anxiety in final models (Block 3).

Independent Variable	Father-Reported Depression					Mother-Reported Depression					Father-Reported Anxiety				
	B	SE	β	*t*	*p*	B	SE	β	*t*	*p*	B	SE	β	*t*	*p*
MH History	**0.69**	**0.25**	**0.34**	**2.76**	**0.009**						**0.60**	**0.25**	**0.32**	**2.36**	**0.023**
Household Income	−0.22	0.37	−0.08	−0.60	0.555	−0.97	0.82	−0.09	−1.18	0.239					
Financial Strain	−0.14	0.28	−0.07	−0.49	0.628	0.60	0.75	0.06	0.80	0.428	−0.01	0.27	−0.01	−0.04	0.969
Employment Loss						−0.07	0.71	−0.01	−0.10	0.919					
RSE Past Month	**0.48**	**0.19**	**0.37**	**2.53**	**0.015**	0.26	0.52	0.04	0.51	0.613	0.33	0.18	0.27	1.86	0.070
RSE Past Year						0.08	0.32	0.02	0.24	0.814					
Paternal Marital Quality	−0.02	0.02	−0.16	−1.17	0.247						−0.02	0.02	−0.12	−0.85	0.399
Maternal Marital Quality						**−0.11**	**0.05**	**−0.16**	**−2.25**	**0.026**					
Maternal Social Support						0.01	0.02	0.02	0.21	0.837					
Maternal Depression						**1.50**	**0.56**	**0.32**	**2.68**	**0.008**					
Maternal Anxiety						0.51	0.56	0.11	0.91	0.365					

*Note.* MH = mental health; RSE = recent stressful events. Shaded cells represent all mother-reported variables (e.g., RSE past month represents mothers’ reports of their own stressful events in the past month). Bolded text indicates the variables that remained significant in the final models.

## Data Availability

The data presented in this study are available on request from the corresponding author. The data are not publicly available due to ethical approvals.
